# Advances in understanding of angioimmunoblastic T-cell lymphoma

**DOI:** 10.1038/s41375-020-0990-y

**Published:** 2020-07-23

**Authors:** Shigeru Chiba, Mamiko Sakata-Yanagimoto

**Affiliations:** Department of Hematology, Faculty of Medicine, University of Tsukuba, Tsukuba, Ibaraki 305-8575 Japan

**Keywords:** T-cell lymphoma, Cancer genetics

## Abstract

It has been nearly half a century since angioimmunoblastic T-cell lymphoma (AITL) was characterized in the early 1970’s. Our understanding of the disease has dramatically changed due to multiple discoveries and insights. One of the key features of AITL is aberrant immune activity. Although AITL is now understood to be a neoplastic disease, pathologists appreciated that it was an inflammatory condition. The more we understand AITL at cellular and genetic levels, the more we view it as both a neoplastic and an inflammatory disease. Here, we review recent progress in our understanding of AITL, focusing on as yet unsolved questions.

## Introduction

The cancer microenvironment is closely associated with inflammation and the immune response. In lymphoid malignancies, a tumor arises in tissue that was once part of the immune system, and thus, inflammatory/immune cells constitute a more dominant component of the microenvironment than in other malignancies.

Angioimmunoblastic T-cell lymphoma (AITL) exemplifies a neoplasm characterized by intense inflammatory and immune reactions, as evidenced by its clinical, pathologic, cellular, and biologic properties. Because tumor cells phenotypically resemble T follicular helper (Tfh) cells [1–3], they are considered to function similarly to some extent to nonneoplastic Tfh cells seen in reactive follicular hyperplasia. However, follicles are not hyperplastic but are rather depleted or destroyed in vast majority of AITL cases.

## Stepwise understanding of AITL—historic overview

In the early-mid 1970’s, different groups proposed what turned out to be a fundamentally identical disease based on observations of independent patient cohorts, namely, a condition variously described as immunodysblastic disease [4], angioimmunoblastic lymphadenopathy with dysproteinemia (AILD) [5], or immunoblastic lymphadenopathy (IBL) [6]. All groups initially proposed that the disease was a nonneoplastic, hyperimmune reaction. Early on, however, investigators recognized that the condition was marked by a broad histologic spectrum and speculated that some patients’ disease might be neoplastic, although they could not make the distinction. Later, a proportion of AILD/IBL cases were diagnosed as malignant lymphoma of either B-cell [7] or T-cell [8] origin. By the late 1980s, the consensus was that most cases described as AILD/IBL were T-cell lymphoma [9, 10]. At the same time, accurate diagnosis of AILD/IBL was facilitated by staining of follicular dendritic cells (FDCs) with anti-CD21 and -CD23 antibodies [11].

In 1994 the term “angioimmunoblastic T-cell lymphoma” was introduced in the Revised European and American Classification of Lymphoid Neoplasms [12]. WHO classifications [13–15] followed this designation, although debate continued until the 2000s over whether true nonneoplastic AILD/IBL exists and whether it represents a premalignant state [13].

Such confusion was in part attributable to uncertainty over tumor cell identity. In the 1990s a consensus was gradually reached that “clear cells” rather than “immunoblasts” represented neoplastic cells [16]. By the early 2000s, important insight had been gained by the discovery of expression of CD10 [17] and C-X-C motif chemokine ligand 13 (CXCL13) [1, 2] in clear cells. Global gene expression patterns seen in whole AITL tissues and isolated tumor cells were then shown to resemble those seen in Tfh cells [3]. These findings confirmed the idea that Tfh cells are the normal cellular counterparts of AITL tumor cells [14]. It is noteworthy that Tfh cell physiology was defined and characterized in parallel [18, 19].

A 2011 report of frequent somatic mutations in the *tet methylcytosine dioxygenase 2* (*TET2)* gene provided insight into AITL genetics [20], as did discovery soon after of recurrent somatic mutations in *DNA methyltransferase 3A* (*DNMT3A)* [21] and *isocitrate dehydrogenase 2* (*IDH2)* [22]. Interestingly, these three genes encode enzymes functioning in epigenetic regulation [23–25] and were originally identified as recurrent mutations in acute myeloid leukemia (AML) and other myeloid malignancies [26–28]. The AITL disease-defining G17A mutation in *ras-homology family member A* (*RHOA)*, *RHOA*^*G17V*^, was identified in 2014 [29, 30]. In AITL, *TET2*, *DNMT3A*, *IDH2*, and *RHOA*^*G17V*^ mutations are seen at around ~80% [29, 31, 32], 20–40% [29–32], 20–30% [22, 29, 31–33], and 50–70% [29–32, 34, 35] of the cases, respectively, followed by mutations in several T-cell receptor (TCR)-related genes, such as *CD28* and *phospholipase C gamma 1* (*PLCG1)* [31, 34, 36]. Of the former four, all but *IDH2* are mutated at similar frequencies in AITL, follicular T-cell lymphoma (FTCL), and in a subset of lymphomas classified by the 2008 version of the WHO classification as peripheral T-cell lymphoma, not otherwise specified (PTCL-NOS) [14]. That subset corresponded to PTCL-NOS with Tfh phenotype [29, 32, 34, 37]. Based on these discoveries, a new umbrella category, namely, “AITL and other nodal T-cell lymphomas of Tfh origin” (hereafter, designated as Tfh lymphomas), was proposed in the 2016 WHO classification to include three diseases, namely, AITL, FTCL, and newly-defined nodal PTCL with Tfh phenotype (nPTCL-Tfh) [15]. In that classification, PTCL-NOS was defined as excluding nPTCL-Tfh. However, diagnosis was not based on this new classification in most of the literature cited here. Here, when we refer to “PTCL-NOS”, we include nPTCL-Tfh, which should largely overlap with PTCL-NOS with Tfh gene expression profiles (GEP) (PTCL-NOS-Tfh) [33, 38].

## AITL incidence—regional differences

The International T-Cell Lymphoma Project (ITCLP) analyzed 1153 PTCL cases (excluding leukemic and cutaneous types and inappropriately diagnosed cases from an original total of 1314) collected from Europe [*n* = 450, 34.2%], North America [*n* = 332, 25.3%], and Asia [*n* = 532, 40.5%]. In that analysis, AITL accounted for 21.1% of PTCL [39], making it the second most frequent PTCL after PTCL-NOS (29.5%). This report confirmed anticipated variations by geographic region for several subtypes such as adult T-cell leukemia/lymphoma (ATLL), extranodal NK/T-cell lymphoma, nasal type (ENKTL), and enteropathy-associated T-cell lymphoma. Unexpectedly, apparent regional variations were also observed in AITL and PTCL-NOS, which accounted for 28.7% and 34.3% of PTCL in Europe, 16.0% and 34.4% in North America, and 17.9% and 22.4% in Asia, respectively. In Asia, ATLL caused by human T-cell leukemia virus type 1 infection (particularly in Japan) and ENKTL closely associated with Epstein–Barr virus (EBV) (particularly in continental east Asia) were markedly overrepresented, which lowered frequencies of other subtypes. However, there remained differences that could not be explained solely by ATLL and ENKTL incidence, as described in the next paragraph.

AITL was recently reported to account for 36.1% of PTCL (excluding cutaneous types) in a combined cohort of 2046 PTCL cases in France, making it more frequent than PTCL-NOS (26.9%) [40]. These incidences are apparently discordant from those reported in the European cohort by the ITCLP. Such discordance could be explained by selection bias, choice of diagnostic tools used at different times of analysis, or differences in 2001 and 2008 WHO classifications on which the ITCLP and French studies, respectively, were based. True geographic variations within Europe have additionally been discussed, particularly with relevance to the Swedish national registry data (2000–2009), in which AITL was diagnosed only in 14% while PTCL-NOS was diagnosed in 29% of non-cutaneous and non-leukemic PTCL (*n* = 755) [40].

**Fig. 1:**
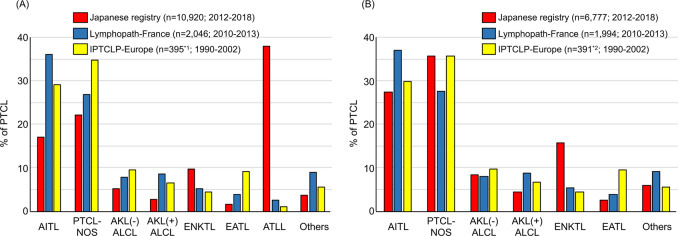
Relative frequencies of non-cutaneous PTCLs. IPTCLP International PTCL Project, ALK anaplastic lymphoma kinase, ALCL anaplastic large cell lymphoma, EATL enteropathy-associated T-cell lymphoma. *1, calculated as 87.8% (frequency of PTCL and ENKTL after excluding non-PTCL cases in IPTCLP) of 450 (number of cases registered in Europe in IPTCLP). *2, calculated excluding ATLL from *1. **a** Frequencies including ATLL. **b** Frequencies excluding ATLL.

In Japan, a national registry for hematologic malignancies based on the 2008 WHO classification was begun in 2012. To compare the incidences of AITL and PTCL-NOS in Japan with those in others, we looked into this registry data with the permission by the Japanese Society of Hematology (Fig. 1). In the 7 years between 2012 and 2018, 11 403 (10 920 non-cutaneous) PTCL cases were registered (Supplementary Table 1). The most frequent diagnosis was ATLL (*n* = 4143; 37.9% in non-cutaneous types), followed by PTCL-NOS (*n* = 2424; 22.2%), AITL (*n* = 1855; 17.0%), ENKTL (*n* = 1063; 9.7%), and ALK(−) ALCL (*n* = 567; 5.2%). As noted, extremely high incidence of ATLL in Japan hampers comparison with other cohorts. If ATLL were excluded, the incidence of AITL and PTCL-NOS would be 27.4% and 35.8%, respectively, figures comparable to the European cohort by the ITCLP (Fig. 1). This factor should be considered in discussions of regional differences in AITL incidence.

## Prognostic indicators

Clinical features of AITL include generalized lymphadenopathy with systemic inflammation/immune-related symptoms/signs, developing most often in the elderly. In five large cohort studies (*n* = 246, 77, 157, 207, and 243, respectively), major clinical parameters of patients were similar: median age (62–67 years), Ann Arbor stage III/IV (81–98%), B symptoms (60–77%), and anemia (51–65% after exclusion of one study with undefined hemoglobin levels) [40].

Five-year overall survival (OS) and event/progression/failure-free survival were 32–41% [40] and 18–38% [41–43], respectively. In the French study (*n* = 157) in which patients were chosen from two prospective clinical trials, a plateau in both OS and EFS was observed around 6 years (7-year OS and EFS, 29% and 23%, respectively) [41]. Plateaus were unclear in other studies, although 7-year OS and PFS were relatively high at 35% and 26%, respectively, in the Japanese retrospective study [42]. In short, the first third dies within a year, the second third between 1 and 5 years, and the last third survives longer. AITL has been described as fatal, but at the same time, long-term survivors have been reported since the first papers were published in the 1970s.

Biomarkers indicative of prognosis have been long sought. Among clinical parameters, high international prognostic index and prognostic index for PTCL-U scores, plus a low platelet count are reproducibly predictive of poor prognosis to some degree [41–43]. No pathologic parameters have been shown to be prognostic indicators, including the presence or absence of clear cells, which are neoplastic but undetectable in ~30% of samples diagnosed as AITL [40]. EBV status in AITL cases is also controversial. In a study evaluating the efficacy of combining rituximab with CHOP, the presence of EBV DNA in the circulation was correlated with the number of EBV^+^ cells in tissues, based on in situ hybridization (ISH) for the EBV-encoded small RNA (EBER; EBER-ISH) in lymph nodes, and with shorter PFS [44]. Others, however, report that the presence of EBV^+^ cells does not alter prognosis [41, 42]. A prognostic model has also been proposed based on gene expression signatures [45]. Focusing on 34 genes whose expression are significantly correlated with clinical outcomes, three functional scores with B-cell, monocytic, and p53-induced signatures have been validated. Among these, a B-cell-associated signature predicted a favorable outcome, while the other two were associated with poorer outcomes. It will be of interest to determine whether the B-cell-associated signature predicts long-term (>5 years) survivors, who might not need aggressive treatments such as autologous transplantation at upfront settings [46].

## Pathology and the Tfh reaction

Typical AITL lymph nodes (pattern III, roughly 80% of AITL) [17, 47] show complete structural effacement. Infiltrating cellular components include clear cells, blastic cells, and arborizing vessels composed of high endothelial venules (HEVs), which have been identified morphologically in 69%, 94%, and 98% of AITL, respectively, based on hematoxylin–eosin staining [40]. Other inflammatory cells are also present, such as small lymphocytes, usually with a normal CD4^+^ and CD8^+^ cell ratio [48], eosinophils, macrophages, and plasma cells. Clear cells correspond to neoplastic cells, but morphologically identifiable clear cells are absent in ~30% of AITL. Biopsied tissues can also exhibit EBV^+^ cells, which are found in 66–91% [37, 40–42, 49] of AITL. Immunostaining reveals the presence of increased FDCs in 93% [40] of cases. Quantity and distribution of all of these cells can differ, resulting in highly significant variation [47, 50]. In addition, there are two nonclassical types of AITL: AITL with hyperplastic follicles (pattern I) and AITL with depleted follicles (pattern II) [17, 47, 50]. Immunohistochemically, CD3, CD4, and CD5 are positive in most cases (100%, 90–95%, and 85–95%, respectively) [41, 42]. CD7 is aberrantly downregulated in many PTCLs [51] and not detectable in 50–70% of AITL cases [41, 42]. AITL neoplastic cells frequently lack cell surface CD3 [51, 52].

Among Tfh cell markers, AITL specimens stain positively for CXCL13, programmed cell death-1 (PD1/PDCD1/CD279; hereafter PD1), CD10, B-cell lymphoma 6 (BCL6), and inducible T-cell co-stimulator (ICOS) in 76–100%, 62–100%, 30–89%, 62-91%, and 98% of cases, respectively [37, 40–42, 49]. It is noteworthy that BCL6 expression in clear cells in AITL had been documented even before Tfh cells were characterized [16, 53]. It is also of interest that a fraction of functional Tfh cells expresses CD10 in normal secondary lymphoid organs [54], although CD10 was initially thought to be aberrantly expressed in AITL neoplastic cells [17]. Based on these and other observations, the 2016 WHO classification requires that Tfh lymphomas be diagnosed by positive immunostaining for at least 2 (ideally 3) of the following 7 antigens: CD10, BCL6, PD1, CXCL13, C-X-C motif chemokine receptor 5 (CXCR5), ICOS, and signaling lymphocytic activation molecule-associated protein (SAP) [15].

**Fig. 2:**
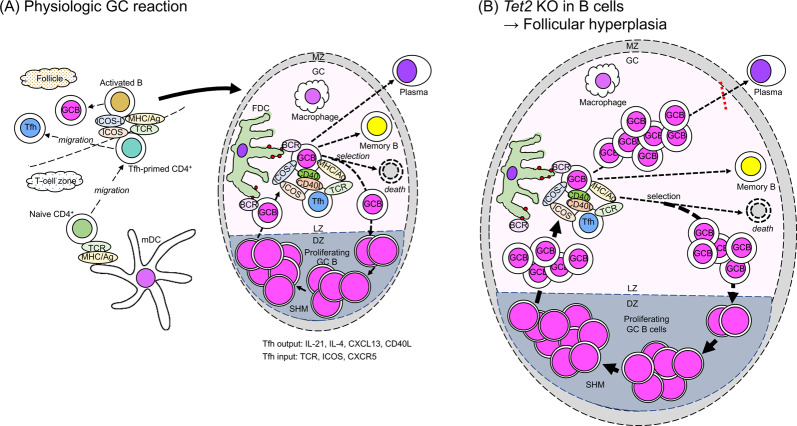
Physiologic and non-physiologic germinal center reactions. (**a**) Germinal center reaction in physiologic condition. Both Tfh-primed cells and B cells together form the GC, where GC B cells begin proliferating at the dark zone and undergo somatic hypermutation. In the light zone, FDCs participate in the network and contribute to selection of affinity-matured GC B cells. In the light zone, GC B cells make one of the three choices: re-entry to the dark zone, differentiation into plasmablast/plasma cells, or differentiation into memory B cells. For all of these processes, CD40 ligand (CD40L) expressed on the Tfh cell membrane and interleukin-21 (IL-21), IL-4, and CXCL13 secreted by Tfh cells play important roles in GC B-cell activation. Engagements of MHC class II, CD40, and ICOS ligand on GC B cells with the TCR, CD40L, and ICOS on Tfh cells, respectively, are of particular importance in terms of direct cell-to-cell contact. Also see the text for additional explanations. (**b**) Germinal center reaction in the presence of *Tet2* disruption in B cells shown in mice. Follicular hyperplasia is caused by impaired exit of GC B cells from the GC light zone. Tfh follicular helper T cell, GCB germinal center B cell, activated B activated B cell, Tfh-primed CD4^+^ Tfh-primed CD4^+^ T cell, naive CD4^+^ naive CD4^+^ T cell, memory B memory B cell, mDC myeloid dendritic cell, FDC follicular dendritic cell, HSC hematopoietic stem cell, Th1 T helper 1 cell, eosino eosinophil. ICOSL ICOS ligand, MHC/Ag antigen presented on major histocompatibility complex, TCR T-cell receptor, CD40L CD40 ligand, BCR B-cell receptor, VEGF vascular endothelial growth factor. GC germinal center, LZ light zone, DZ dark zone, BM bone marrow, LN lymph nodes. SHM somatic hypermutation, mut mutation. Red closed circles indicate antigen localized on FDC.

Understanding of Tfh cell function in germinal center (GC) formation and reactions has greatly expanded over the past two decades; in particular, progress in the last decade has been swift due to identification of BCL6 as a master transcription factor for Tfh cells [55–57]. Priming of naïve CD4^+^ T cells to Tfh cells is the first step of GC formation and thus of all humoral immunity. Complex activities involving multiple factors then proceed, including interactions between Tfh and B cells and between B cells and FDCs. (See a recent review [19] for a comprehensive summary of these processes.) In brief (Fig. 2a), interaction of naïve CD4^+^ T cells with dendritic cells (DCs) at the T-cell zone governs the choice toward either a Tfh cell fate based on BCL6 expression or a non-Tfh cell fate marked by expression of B lymphocyte-induced maturation protein-1 [55–58]. Expression of the co-stimulatory molecule ICOS by DC-activated naïve CD4^+^ T cells is an early event in Tfh priming [58]. Tfh-primed cells migrate to the T/B border and start interacting with activated antigen-specific B cells [19]. It is suggested that ligation between PD1 and PDL1 controls Tfh cell positioning [59].

**Fig. 3:**
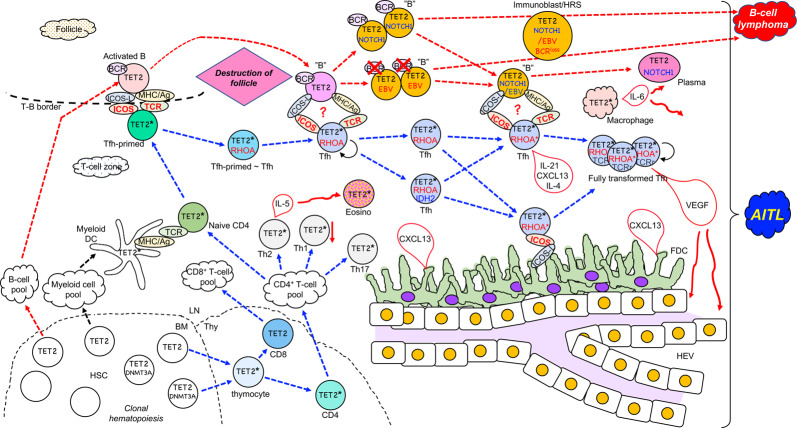
Schematic model of AITL generation. In the bone marrow (BM), somatic mutations in *TET2* (or *TET2* plus *DNMT3A*; marked as TET2 and DNMT3A) result in clonal hematopoiesis. *TET2* alone or *TET2* plus *DNMT3A* mutated hematopoietic stem cells (HSC) can give rise to thymocytes. *TET2* plus *DNMT3A* mutated HSC generate more CD4^+^ T cells than CD8^+^ T cells. TET2^*^ indicates *TET2* mutation alone or *TET2* plus *DNMT3A* mutations. *TET2* alone or *TET2* plus *DNMT3A* mutated naive CD4^+^ T cells are primed to Tfh cells by the contact with myeloid DC cells, and migrate to the T-B border. The *TET2* mutation (or *TET2* plus *DNMT3A* mutations)-carrying Tfh-primed cells contact with the *TET2*-mutated activated B cells, then acquire the *RHOA*^*G17V*^ mutation (RHOA) before or after differentiation into Tfh cells (Tfh). These Tfh cells should further interact with B cells (“B”) derived from activated B cells at the follicle-destroyed lymph nodes. The Tfh cells carrying *TET2* (or *TET2* plus *DNMT3A*) plus *RHOA* mutations may further acquire the *IDH2* mutation (IDH2). *RHOA* mutation alone or *RHOA* plus *IDH2* mutations are designated as RHOA^*^. Ultimately, mutations in TCR-related genes (TCRr) are acquired. *TET2*-mutated B cells are infected with EBV and/or acquire mutations in genes such as *NOTCH1*. These Tfh cells and B cells with individually accumulated mutations still contact and activate each other through ICOS-L and ICOS ligation and still unknown mechanisms (designated as “?”). Some of the B cells take a morphology as immunoblasts or HRS cells, and B-cell lymphoma arises. See the text for the further scenario. LN lymph nodes, Thy thymus. HSC hematopoietic stem cell, naive CD4^+^ naive CD4^+^ T cell, Tfh-primed Tfh-primed CD4^+^ T cell, Tfh follicular helper T cell, activated B activated B cell, Th17 T helper 17 cell, Th1 T helper 1 cell, Th2 T helper 2 cell, eosino eosinophil, plasma plasma cell, FDC follicular dendritic cell, HEV high endothelial venule. ICOSL ICOS ligand, MHC/Ag antigen presented on major histocompatibility complex, TCR T-cell receptor, BCR B-cell receptor, VEGF vascular endothelial growth factor.

Tfh activity is closely associated with numerous pathologies, including infectious, allergic, autoimmune, atherosclerotic, and neoplastic disease [19]. AITL (other than pattern II), however, is unique because the physiologic GC reaction described above is completely abrogated. Neoplastic Tfh cells are hypothesized to function in disease initiation and development. Understanding AITL pathology requires defining stage(s) of Tfh development and activity that differ between physiologic and neoplastic Tfh cells (see Figs. 2, 3).

## AITL genetics, Tfh cell specification and differentiation, and AITL modeling

AITL is characterized by several recurrent mutations, many analyzed molecularly in mice. Surprisingly, functionally distinct mutations respectively promote Tfh cell specification or differentiation. Thus, further understanding is required of how these changes lead to AITL/Tfh lymphoma development. What follows is a list of some of the most predominant mutations.

### *TET2* mutations

*TET2* is the most frequently mutated gene in AITL: *TET2* somatic variations are seen in ~80% of patient specimens, with many exhibiting 2 or more *TET2* mutations [29, 31, 32]. *TET2* encodes a dioxygenase that transfers oxygen to 5-methylcytosine (5mC) in DNA and converts it into 5-hydroxymethylcytosine (5hmC) and further sequentially to 5-formylcytosine (5-fC) and 5-carboxylcytosine (5-caC) [23, 60]. *TET2* mutations promote loss-of-function of the TET2 enzyme [61] and this is true for AITL [20].

To model *TET2* mutations in animals, several groups have analyzed mice with *Tet2*-disruption or -downregulation in either germline or whole blood cells. In general, mutant mice develop both chronic myelomonocytic leukemia-like disease [20, 62] and mature T-cell lymphoma with Tfh phenotype [63]. Skewed Tfh cell differentiation in splenic CD4^+^ T cells prior to lymphoma development has also been reported [63]. In this report, mice died at a median survival period of 67 weeks, developing T-cell lymphoma with Tfh-like features. It has also been demonstrated that *Bcl6* is upregulated by *Tet2* downregulation via hypermethylation at a *Bcl6* intronic silencer region [63].

TET2 loss-of-function could affect any stage of T-cell development [20, 63]. One critical stage may be that of naive CD4^+^ cells, in which *TET2* downregulation would enhance *BCL6* expression and initiate skewed differentiation toward Tfh cells (Fig. 3).

### *RHOA*^*G17V*^ mutations

*RHOA* mutations, most of which are missense and result in Gly to Val substitution at amino acid 17, are found in 50–70% of AITL [29–32, 34, 35]. *RHOA* mutations are seen at relatively high frequencies in other lymphomas such as ATLL [64] and pediatric Burkitt’s lymphoma [65], and diffuse-type gastric cancer [66]. In these neoplasms, however, mutations occur at hotspots different from Gly17. *RHOA*^*G17V*^ mutations are reportedly confined to a microdissected PD1^+^ cell fraction enriched for cells with Tfh features, which are absent in the CD20^+^ cell fraction enriched for B cells [67], strongly indicating that *RHOA*^*G17V*^ mutations are acquired after divergence of the T-cell fate from the B-cell fate. Wild-type RHOA functions as a small GTPase, a function lost in RHOA^G17V^ due to loss of nucleotide binding capacity [29, 30, 35]. Instead, RHOA^G17V^ acquires a neo-function, namely, binding capacity to VAV1 protein [68], which normally functions as a guanine nucleotide exchange factor (GEF) and GEF-independent adaptor in the TCR signaling pathway [69, 70]. Both GEF-dependent and -independent functions are regulated by tyrosine phosphorylation [68, 71, 72]. TCR-associated FYN and LCK are among candidate tyrosine kinases responsible for VAV1 phosphorylation [71, 73, 74]. Studies in Jurkat cells show that RHOA^G17V^ increases TCR signaling through enhanced VAV1 phosphorylation at Tyr174 [68]. In vivo, this likely results in stronger TCR signaling input to RHOA^G17V^-expressing naive CD4^+^ T cells, which can contribute to preferential commitment to Tfh rather than non-Tfh lineages upon engagement with DCs [75].

Multiple mouse models established by retroviral transfer [76], knock in [77], or transgenic [78] techniques have been used to assess the effects of expression of *RHOA*^*G17V*^ or its murine version *Rhoa*^*G17V*^. Analysis of *Rhoa*^*G17V*^ knock in and *RHOA*^*G17V*^ transgenic models in which either is driven by the CD4 promoter show that both *Rhoa*^*G17V*^ and *RHOA*^*G17V*^ per se induce Tfh specification and differentiation in spleen [77, 78]. In the presence of Rhoa^G17V^, Icos expression is induced in vitro by stimulation of CD4^+^ cells with irradiated antigen-presenting cells and anti-CD3 antibody [77]. ICOS functions as a co-stimulatory molecule and a migration receptor for Tfh cells, and its signaling is essential at both the initial step of Tfh specification [58, 79] and in maintenance of Tfh phenotypes [80]. *RHOA*^*G17V*^ transgenic mice produce antibodies to double-stranded DNA and exhibit autoimmune-like phenotypes, such as skin rash and renal immune complex deposition [78]. RHOA^G17V^/Rhoa^G17V^ expression likely perturbs many other regulatory programs in CD4^+^ T cells; for example, it enhances CD4^+^ T-cell proliferation and reduces differentiation into T helper type 1 (Th1) cells [77]. The effect of RHOA^G17V^/Rhoa^G17V^ expression in CD4^+^ T cells on differentiation to regulatory T cells remains controversial [77, 78].

Taken together, the presence of RHOA^G17V^ enhances both TCR and ICOS signaling possibly through independent mechanisms, specifies Tfh lineage, maintains Tfh phenotypes, and provokes autoimmune reactions, all reminiscent of outcomes associated with AITL and other Tfh lymphomas (Fig. 3). These outcomes may explain why *RHOA*^*G17V*^ mutations are genetically selected specifically in Tfh lymphomas.

### Combined *Tet2* loss-of-function and *RHOA*^*G17V*^ or *Rhoa*^*G17V*^ expression—an AITL model

*Tet2* disruption or *RHOA*^*G17V*^/*Rhoa*^*G17V*^ expression independently induces Tfh differentiation, as discussed in the two previous subsections. Although T-cell lymphoma with Tfh phenotypes is seen following *Tet2* downregulation in mice, histology of lymphoma tissues does not resemble AITL [63]. It is thus of great interest to assess phenotypes in mice with combined *Tet2* disruption and *RHOA*^*G17V*^ or *Rhoa*^*G17V*^ expression. So far, using independent approaches to engineer such mouse models, three groups have reported development of lymphoma with Tfh features, and lymphoma tissue histology more resembling classical AITL, including increased numbers of blood vessels [77, 78, 81].

Comparison of these models provides insight into mechanisms underlying AITL development. In the first model [77], *Tet2* disruption and knocked-in *Rhoa*^*G17V*^ expression were both driven by the mouse *CD4* promoter using the Cre-lox system (CD4-Tet2-Rhoa), confining both mutations to the T-cell compartment. In the second model [78], *Tet2* disruption was driven by the *VAV1* promoter-Cre and transgenic *RHOA*^*G17V*^ expression by the mouse *CD4* promoter (VAV1-Tet2/CD4-RHOA); thus, *Tet2* is disrupted in all hematopoietic cells, while *RHOA*^*G17V*^ expression is confined to T cells. In the third model [81], *Tet2* disruption was driven by the *Mx* promoter-Cre following pIpC injection and transgenic *RHOA*^*G17V*^ expression was driven by the human *CD2* promoter (Mx-Tet2/CD2-RHOA); thus, *Tet2* disruption and *RHOA*^*G17V*^ expression are controlled in largely the same manner as in the VAV1-Tet2/CD4-RHOA model.

In the first model, AITL-like lymphoma did not naturally develop: bone marrow transplantation from Tet2-Rhoa-CD4 mice to syngenic mice followed by immunization with sheep red blood cells (SRBCs) was required for development of AITL-like lymphoma in recipients [77]. By contrast, mice in the third model showed development of AITL-like lymphoma [81]. These outcomes suggest that, in patients, *TET2* loss-of-function mutations in non-T-cell microenvironmental cells may be an important inducer of AITL in the context that *RHOA*^*G17V*^ mutations are present in T cells. *TET2* loss-of-function mutations in T cells might also contribute to AITL establishment in the presence of *RHOA*^*G17V*^ mutations in T cells, if the host is exposed to strong immunization. SRBC immunization induces GC hyperplasia and, as described below, *TET2* mutations in B cells may also contribute to GC hyperplasia [82]. Therefore, induction of GC hyperplasia could be a critical event in promoting AITL development.

In summary, both *TET2* and *RHOA*^*G17V*^ mutations can enhance Tfh differentiation from naïve CD4^+^ T cells. Signaling inducing GC hyperplasia is necessary and sufficient for AITL establishment in the context of enhanced Tfh differentiation. *TET2* mutations alter transcription programs in both T and B cells, which may activate signaling that induces GC hyperplasia. Nevertheless, gaps remain in our understanding of GC hyperplasia (Fig. 2b) versus AITL (Fig. 3) pathology.

### *IDH2*^*R172*^ mutations

In AITL, *IDH2*^*R172*^ mutations occur at a frequency of 20–30% [22, 29, 31–33], while *IDH1*^*R132*^ and *IDH2*^*R140*^ mutations, which recur in AML [25, 83], are very rare. IDH enzymes normally convert isocitrate to α-ketoglutarate (αKG); AML/AITL-associated mutant IDH proteins acquire a neo-function leading to production of the oncometabolite 2-hydroxyglutarate (2HG) [25, 83, 84]. 2HG inhibits iron- and αKG-dependent dioxygenase activity [25]. *TET2* and *IDH1/2* mutations are found in a mutually exclusive manner in AML, and *IDH1/2* mutations in AML promote a DNA hypermethylation signature similar that is associated with *TET2* mutations. Therefore, TET2 is considered an important target of 2HG in AML [25].

In AITL, in contrast, *IDH2*^*R172*^ mutations are identified almost exclusively in samples that also harbor *TET2* mutations [22, 29, 31–33], and cells in those samples generally show markedly increased H3K27me3 levels plus hypermethylation of promoter regions genome wide. Thus, histone demethylases are likely important targets of 2HG generated by IDH2^R172S/M/G/K^ in AITL [32], as has been shown in glioma marked by IDH1^R132H^ and IDH2^R172K^ mutations [85].

In a mouse model, expression of IDH2^R172K^ but not I IDH1^R132H^ mutants increases 2HG levels in T cells, corresponding to the specificity of *IDH2*^*R172*^ mutations with T-cell lymphpoma [86]. Microdissection of clinical AITL samples has identified that *IDH2*^*R172*^ mutations are unique to PD1^+^ cells [67] and correspondingly, IDH2^R172^-specific antibody reacts specifically with ICOS^+^ cells [86]. It is thus thought that acquisition of *IDH2*^*R172*^ mutations occurs shortly before or after Tfh commitment.

Importantly, among Tfh lymphomas, *IDH2*^*R172*^ mutations are mostly confined to AITL and are rare in FTCL [37] and PTCL-NOS with Tfh phenotype or GEP [31, 33, 37, 38, 40], unlike recurrent mutations in *TET2*, *DNMT3A*, and *RHOA*, which are common to all types of Tfh lymphomas. *IDH2*^*R172*^ mutation-positive AITL cases define a discrete subpopulation in both GEP [32, 33] and genetic profiles [33]. *IDH2*^*R172*^ mutation-positive AITL cases generally show downregulation of Th1 differentiation-associated genes and upregulation of Tfh-associated genes [33]. Furthermore, *IDH2*^*R172*^ mutation-positive samples exhibit increased expression of vascular endothelial cell growth factor (VEGF) [33], which may be associated with increased vascularization seen in AITL and account for why this mutation is unique to AITL-specific histology. Genetically, a gain of chromosome 5 (Chr5) uniquely characterizes AITL and is seen in 43% (15/35) of samples [33]. *IDH2*^*R172*^ mutations are also associated with Chr5 gain (10/14 chr5 gain-positive cases vs. 4/18 negative cases). *Interleukin-4* (*IL-4*) is localized on Chr5 and its expression increases in cases marked by Chr5 gain [33], an outcome corresponding to accelerated Tfh differentiation observed in *IDH2*^*R172*^ mutation-positive cases.

*IDH2*^*R172*^ mutations may be associated with enhanced Tfh features and histopathologic characteristics of AITL such as increased blood vessels, through changing epigenetic regulation and gene expression signatures (Fig. 3).

### TCR-related gene variations

Variations in TCR-associated genes, such as *PLCG1*, *CD28*, *VAV1*, and *FYN*, are seen in approximately half of AITL cases [30, 33, 34]. Moreover, structural variations such as gene fusions have been identified for *CD28*, *VAV1*, and *ITK* in AITL samples [87, 88]. Given that strong TCR signaling in naïve CD4^+^ T cells dictates the choice toward a Tfh fate [75], these mutations may contribute to AITL development. However, most of them (with the exception of particular mutations and fusions of *CD28* [33, 88]) are not specific to AITL or Tfh lymphomas but occur equally or even with greater frequency in PTCL-NOS [38] or ATLL [89]. Thus, their consequences likely differ significantly from mutations in *TET2*, *RHOA*^*G17V*^, and *IDH2*^*R172*^ with respect to AITL. Variations in TCR signaling pathway genes may broadly influence T-cell activities, such as proliferation (Fig. 3), resistance to apoptosis, migration, or cytokine production, and generally contribute to development of multiple PTCLs. Abnormal RHOA^G17V^-VAV1 signaling is seen in AITL and nPTCL-Tfh [68], but *VAV1* mutations/fusions may play different roles in PTCLs other than Tfh lymphomas.

### *DNMT3A* mutations

*DNMT3A* mutations in AITL occur at a frequency of 20–38.5% [29–32], a frequency similar to that seen in PTCL-NOS with Tfh phenotype/GEP [29, 32, 33, 38]. However, two recent studies report frequencies of *DNMT3A* mutations in PTCL-NOS categorized as PTCL-NOS-GATA3 or PTCL-NOS-TBX21 [45] as <15% [33, 38], indicating aberrant accumulation of these mutations in Tfh lymphomas. Currently, there is no evidence that *DNMT3A* mutations directly perturb Tfh cell specification or differentiation. Interestingly, most *DNMT3A* mutations in Tfh lymphomas occur together with *TET2* mutations [29–32]. *Tet2*/*Dnmt3a* double-knockout mice show synergistic effects on expression of lineage-specifying transcription factor genes via epigenetic mechanisms [90]. Cooperation between *Tet2* knockout and *DNMT3A*^*R882H*^ expression is also shown by the transplantation experiment with the *Tet2-null* hematopoietic stem/progenitor cells retrovirally expressing *DNMT3A*^*R882H*^ [91]. In this report, serial transplantation results in development of AITL-like disease. It will be of interest to determine whether *DNMT3A* mutations actually enhance the effect of *TET2* mutations on promoting Tfh cell differentiation. *DNMT3A* and *TET2* mutations are often co-identified in clonal hematopoietic cells in Tfh lymphoma patients [21, 29, 67]. Similar to the profile of *DNMT3A* mutations found in clonal hematopoiesis in healthy individuals, mutations observed in Tfh lymphoma patients do not accumulate in the *DNMT3A* methyltransferase domain, and hotspot R882(H) mutations are less frequent in AITL samples [29, 33, 38]. Overall, *DNMT3A* mutations should be involved in clonal hematopoiesis, but it still remains unclear whether they are directly involved in the Tfh specification or differentiation in Tfh lymphomas (Fig. 3).

### *RC3H1/Roquin* and GAPDH—AITL mouse models not based on patient genetics

Clinical AITL samples do not show variations in the *ring finger and CCCH-type domain 1 (RC3H1/ROQUIN)* [92]; nonetheless, mice heterozygous for the *Roquin* missense allele *Roquin*^*san*^ develop AITL-like lymphoma with an approximately 50% penetrance [93]. Roquin is an RNA-binding ring-type ubiquitin ligase discovered in a mutagenesis screen for autoimmune phenotypes in mice [94]. Homozygous missense mutations (M199R) in *Rc3h1/Roquin* (*san/san*) cause systemic lupus erythematosus-like disease with marked generalized lymphadenopathy. Heterozygous *san/+* mice, which develop asymmetric lymphadenopathy, have been analyzed as a lymphoma model [93]. Wild-type Roquin binds to *Icos* mRNA to promote its degradation, and mutant Roquin encoded by *Roquin*^*san*^ binds to *Icos* mRNA with a higher affinity than does the wild-type protein, leading to slower mRNA decay [95], aberrant expression of Icos on CD4^+^ T cells, and an excessive number of Tfh cells. *san/+* mice show many features of human AITL such as hypergammaglobulinemia [93], and their analysis has contributed to recognition that enhanced ICOS signaling can promote AITL tumorigenesis. There are, however, no reports that ICOS overexpression per se causes AITL/Tfh lymphoma, and thus, it remains uncertain whether other factors downstream of *Roquin*^*san*^ contribute to AITL/Tfh lymphoma in concert with aberrant ICOS expression.

Glyceraldehyde-3-phosphate dehydrogenase (GAPDH), one of ten enzymes functioning in aerobic glycolysis, has additional functions [96, 97]. It was recently reported that mice with GAPDH overexpression in T cells develop Tfh lymphoma with AITL-like features by the age of 18 months or older [98]. GAPDH reportedly activates the non-canonical nuclear factor kappa B (NFκB) pathway, a phenotype seen in these mice. As a consequence, CD4^+^ T cells undergo Tfh differentiation and express PD1. In GC B cells, the non-canonical NFκB pathway is activated through PD1–PDL1 ligation, which likely contributes to AITL-like lymphoma development in GAPDH-overexpressing mice. Non-canonical NFκB signaling is also activated in human AITL [98], although it is uncertain whether PD1–PDL1 ligation triggers such an activation in patients. GAPDH-overexpressing mice have also been used to test efficacy of an inhibitor of NFκB-inducing kinase (NIK), which activates non-canonical NFκB signaling. The efficacy of NIK inhibitors as treatment for AITL should be elucidated in clinical trials.

## Microenvironmental cells derived from clonal hematopoiesis

With age, healthy individuals increasingly exhibit clonal blood cells harboring driver mutations, most frequently in *DNMT3* or *TET2* [99, 100]. Given that disruption of either *Tet2* [20, 62] or *Dnmt3a* [101] in mice enhances HSC self-renewal, comparable loss-of-function mutations likely enhance self-renewal of human HSCs.

In AITL and other Tfh lymphomas, *TET2* (and *DNMT3*) mutations are often (7 of 10 cases by 1 group [20, 21] and 4 of 7 cases by another [29]) identified in normal-appearing bone marrow cells, CD34^+^ cells, and colony-forming blood progenitor cells. These mutations are also reported in multiple lineages of tumor-infiltrating cells by two groups (16 of 17 AITL and PTCL cases by 1 group [67] and 6 of 10 AITL cases by another [102]). Therefore, it is anticipated that most cases of AITL show clonal hematopoiesis, with most carrying either *TET2* or *TET2*/*DNMT3A* mutations. It remains a fundamental question whether *TET2* and *DNMT3A* mutations function in multiple lineages of HSC progeny to drive AITL development. This possibility was discussed in part in the previous comparison of three AITL mouse models and is addressed in greater detail below.

### B cells

Mice mutant in *Tet2* in B cells, when immunized with SRBC, exhibit GC hyperplasia, possibly due to impaired exit of GC B cells from the GC light zone following failure to initiate cell-autonomous gene expression patterns [82]. Thus, GC B cells are retained longer in the GC center and likely have increased opportunities to interact with Tfh cells (Fig. 2b). When typical AITL is initiated, either a single Tfh cell or a CD4^+^ T cell specified to become a Tfh cell due to *TET2* mutations likely acquires a *RHOA*^*G17V*^ mutation and proliferates. Eventually, Tfh cells harboring both mutations interact with *TET2-*mutant activated antigen-specific B cells. Then, among scenarios proposed to explain AITL histopathology, follicles may burst for unknown reasons (Fig. 3). In analysis of B-cell-specific *Tet2*-deficient mice, differentiation of B cells to plasma cells is impaired via downregulation of the gene encoding the transcription factor Blimp-1 [82] (Fig. 2b). However, polyclonal gammopathy is an AITL hallmark. Indeed, plasma cells are often abundant in AITL tissue, and polyclonal plasmacytosis is sometimes seen in AITL patients [103, 104]. Therefore, a plasma cell differentiation impairment, which is seen in mice mutant in *Tet2* in B cells, may be reversed in AITL (Fig. 3). Or, *TET2* deficiency due to somatic mutation in AITL may affect the plasma cell differentiation differently from the *Tet2*-deficient mouse model, in which *Tet2* is disrupted at the B-cell development during embryogenesis.

### DCs and macrophages

DCs and macrophages are among infiltrating myeloid cells in AITL [47]. Tet2 activity resolves inflammation by actively and selectively repressing *Il-6* expression in innate myeloid cells, including DCs and macrophages [105]. In this case, *Il-6* repression is independent of 5-mC dioxygenase activity: Tet2 recruits histone deacetylase 2 at the late phase of inflammation in response to innate immune stimulation [105]. Although this activity is not directly related to AITL development, elevated Il-6 production is seen in a mouse AITL model [81]. That excess IL-6 may function in AITL development and be secreted by DCs and macrophages carrying *TET2* mutations (Fig. 2c).

When cultured with low-density lipoprotein (LDL), macrophages from bone marrow of *Tet2* knockout mice express higher levels of mRNAs encoding chemokines and cytokines such as Cxcl1, Cxcl2, Cxcl3, platelet factor 4 (Pf4), Il-1b, and Il-6 [106]. Indeed, mice with mature myeloid cell-specific *Tet2* deficiency show enhanced atherosclerosis [106]. Macrophage and DC hyperactivity may not be confined to LDL stimulation: some microenvironmental stimulation may recapitulate activity of *TET2-*mutant macrophages and DCs and contribute to AITL development in lymph nodes.

### T cells

A variety of T-cell subsets are also present in the microenvironment, and a substantial proportion of each subset is presumed to carry *TET2* mutations or *TET2* plus *DNMT3A* mutations. As expected, *TET2* mutations are acquired before the *TCRB* rearrangement and multiple *TCRB* clones are produced from a progenitor carrying a single *TET2* mutation [107]. T-cell functions are highly variable depending on the subset and the context. These variations may be further complicated by the *TET2* and *DNMT3A* mutations, details of which remain to be clarified.

## Other genetic or genomic abnormalities seen in B cells in AITL

### EBV infection

In AITL cases with EBV^+^ B cells (which accounts for 66–91% of AITL cases [37, 40–42, 49]), EBER-ISH often detects virus in blastoid B cells or large B cells resembling Hodgkin/Reed–Sternberg (HRS) cells [108, 109]. Monoclonal or oligoclonal immunoglobulin (Ig) gene rearrangement is detected in up to one-third of AITL cases [47]. EBV infection and clonal expansion of B cells in AITL are often associated with each other. Such clonal B-cell growth may be further associated with development of diffuse large B-cell lymphoma (DLBCL) and other B-cell lymphomas, which are sometimes observed concomitantly with AITL (as a composite lymphoma [110]) or at a later phase of AITL. Indeed, tumor cells in B-cell lymphoma developing in association with AITL are often infected with EBV. Interestingly, EBV^+^ B cells reportedly lose functional Ig due to destructive hypermutations in most AITL cases accompanied by EBV^+^ B cells [108], indicating that B-cell proliferation in these cases does not require B-cell receptor (BCR) signaling but rather is driven by a different pathway (Fig. 3).

In de novo DLBCL, the frequencies of *TET2* mutations were reported to be 5/48 (10.4%) and 9/27 (33%), and those of *DNMT3A* mutations 0/48 (0%) and 3/27 (11.1%) (*p* < 0.05 in both comparisons) in EBV^−^ and EBV^+^ DLBCL, respectively [111]. All the three cases with *DNMT3A* mutations had *TET2* mutations, the situation very similar to AITL. *TET2* and *DNMT3A* mutations were the only ones which showed significant positive correlation with EBV positivity, while multiple gene mutations showed positive correlation with EBV negativity in DLBCL. It is thus reasonable to speculate that *TET2* (and *DNMT3A*) mutations cooperate with EBV for DLBCL development, irrespectively of whether it is de novo or associated with AITL.

### B cell-specific somatic mutations in AITL/PTCL-NOS

While HRS-like cells in AITL [109] and tumor cells in DLBCL, which is associated with AITL [112] are often infected with EBV, HRS-like cells in some AITL cases and tumor cells in some AITL-associated DLBCL cases can be EBV-negative. In these cases, it is reasonable to assume acquisition of genetic hits that recapitulate abnormal B-cell activities by EBV infection.

Somatic mutations identified by targeted sequencing in whole AITL and PTCL-NOS tissues were separately analyzed in a CD19^+^ cell fraction enriched for B cells and a PD1^+^ cell fraction enriched for tumor cells, both isolated by microdissection of individual samples [67]. Several mutations in *TET2*, *DNMT3A*, and other genes were identified in both fractions. *RHOA*^*G17V*^ and *IDH*^*R172*^ mutations plus other mutations were exclusively identified in the PD1^+^ cell fraction. Interestingly, patient-specific *NOTCH1* mutations were identified only in the CD19^+^ cell fraction in the three samples. In multiple B-cell malignancies such as chronic lymphocytic leukemia [113], mantle cell lymphoma [114], and DLBCL [115], activating *NOTCH1* mutations are among major drivers. Targets of NOTCH signaling in this context are associated with BCR and cytokine signaling, as well as MYC pathways [116]. *NOTCH1* mutations may be acquired when *TET2* mutation-carrying clonal hematopoiesis cells differentiate into B cells and subsequently may enhance effects of *TET2* mutations. Interactions of B cells harboring *TET2* and *NOTCH1* mutations with Tfh cells harboring *TET2* and *RHOA* mutations likely mediate bidirectional signaling necessary for AITL development (Fig. 3).

B-cell-specific mutations and EBV infection may be important in AITL development. The relationship between the mutations and EBV infection, however, is still not known.

## Non-hematopoietic microenvironmental cells in AITL

### FDCs and HEVs

Increases in FDCs and HEVs are unique to AITL; thus, these two cell types are likely involved in AITL pathophysiology [117]. Both cell types are distinct from other immune cells, which are HSC progeny; FDCs are mesenchymal in origin and HEVs are endothelial.

In a normal GC reaction, FDCs provide antigens to GC B cells at the light zone, an activity required for affinity maturation of GC B cells [118]. GCs cannot be maintained without FDCs [19]. Mechanisms underlying abnormal FDC growth in AITL are not known. FDCs produce abundant IL-21, which may contribute to AITL development. At least in AITL cases with EBV infection, FDCs likely do not provide antigens to B cells, given that proliferation of EBV-infected B cells is independent of BCR signaling [108], as discussed. It is demonstrated that ICOSL-ICOS- phosphatidyl inositol 3-kinase (PI3K) pathway plays an important role for the growth of neoplastic Tfh cells in the AITL mouse model [77]. Because ICOSL is highly expressed in FDCs as well as B cells, activated monocytes, and mDCs [77], one of the potential functions of FDCs in AITL could be to stimulate ICOS in neoplastic Tfh cells.

VEGF overproduction is likely associated with the increase in HEVs, although how HEVs impact AITL establishment is obscure. Typically, HEVs, FDCs, and neoplastic Tfh cells localize near each other [47, 50]. Thus, cell-cell contact among them may be important for AITL establishment and development. Understanding the precise roles of FDCs and HEVs awaits development of new research tools, such as use of single cell analysis (Fig. 3).

## AITL as an “immunodysplastic” syndrome

AITL was originally designated an immunodysplastic disease [4], and “immunodysplastic syndrome (LDS)” was proposed as a name for this disease [119]. The term “dysplastic” appears to have indicated both morphologic and functional features. The former indicated appearance of multiple kinds of abnormal cells in tumorous lymph nodes, including immunoblasts and clear cells (and may be HRS-like cells), as well as increased HEVs and FDCs. “Dysplastic” function was used to describe hypergammaglobulinemia and immunodeficiency. LDS was proposed to emphasize the contrast with myelodysplastic syndrome(s) (MDS). If myeloblasts are regarded as tumor cells in MDS, then low tumor cell burden is common to both MDS and AITL. In both diseases, most cellular components are mature HSC-derived non-tumor cells, including myeloid cells in MDS and both lymphoid and myeloid cells in AITL. These cells, originating from clonal hematopoiesis and often harboring *TET2* mutations, provide an abnormal tumor microenvironment and likely function in development of both diseases.

## Budding of mechanism-based treatments

The prognosis of AITL is generally poor as already discussed, though with some long-term survivors. There have been no satisfactory strategies including a variety of combination chemotherapies.

### Introduction of rituximab

Based on the idea that environmental B cells could be an important contributor for AITL promotion and maintenance, a clinical trial of combination of rituximab and CHOP was performed [44]. In this phase II study (*n* = 25), an overall response rate was as high as 80% but the median response duration was 25 months in patients achieving a complete or unconfirmed complete response. It was thus concluded that the addition of rituximab to the standard CHOP did not show a clear benefit. This result, however, may not exclude the possibility of targeting B cells in the future clinical trials if suitable candidates for combination emerge.

### Immune checkpoint inhibitors

Another one of the interests is immune checkpoint inhibitors. In the vast majority of AITL, the neoplastic Tfh cells express PD1, which may play a role for the Tfh cell migration [59] or NFκB activation in PDL1-expressing B cells [98]. Therefore, the significance of the blockade of PD1–PDL1 ligation in AITL may be different from the usual immune checkpoint inhibition in other neoplasms. A phase II clinical trial testing nivolmab, an anti-PD1 antibody, in patients with PTCL was halted because 4 of 12 patients evaluated in the interim analysis showed hyperprogressive disease (rapid progression within 1 cycle of treatment) [120]. Six of the 12 patients were those with AITL, among whom complete response was achieved in one. Hyperprogressive disease was indeed seen in at least one AITL patient [121]. This could indicate that PD1–PDL1 ligation is suppressing AITL, despite the speculation that PD1 plays a tumor promoting role. Studies in animal models might convey further information.

### Hypomethylating agents

Hypomethylating agents (HMAs), 5-azacytidine (Aza), and decitabine, are approved for the treatment of MDS in many countries. HMAs counterbalance the DNA hypermethylation status caused by mutations in epigenetic regulators, such as *TET2* and *IDH2*. Theoretically, therefore, HMAs can be effective in diseases carrying *TET2* mutations. Actually, Aza is shown to be significantly more effective for MDS with *TET2* mutations than for MDS without [122, 123]. Because the frequencies of *TET2* mutations are much higher in AITL than in MDS, HMAs can be a good candidate as a medicine to treat AITL. Aza alone indeed showed a promising result in a retrospective study [124]; all 12 patients had *TET2* mutations and 9 of the 12 responded to Aza. The response was sustained for >23 months in all the five patients achieving a complete response. The result of a phase II clinical trial designing a combination of oral Aza and romidepsin, a histone deacetylase inhibitor, was also promising for PTCL patients (overall response in 8 of 11; median duration of response not reached at the median follow-up period of 15.3 months), including some with AITL [125].

### Dasatinib

As a mechanism how *RHOA*^*G17V*^ mutations drive AITL, hyperphosphorylation of VAV1 [68] is already described. A multi-kinase inhibitor dasatinib inhibits the Src family tyrosine kinases including FYN and LCK, probable candidates responsible for VAV1 phosphorylation at the downstream of TCR. Based on the results showing the effect of dasatinib on the AITL mouse model, phase I clinical trial of dasatinib in AITL patients were performed and showed a promising result (4 of 5 responded to dasatinib) [81]. The efficacy should be shown in the next-step clinical trials.

### Other possibilities

NIK inhibitors are already referred to [98]. Duvelisib, a PI3Kδ inhibitor that blocks ICOS-PI3K pathway, may be a candidate [77]. Many other possible agents, antibodies, etc. may emerge; they could target neoplastic Tfh cells, environmental cells, and both. Inhibition of cell-to-cell interaction can also be a right target.

## Future perspectives

New technologies, such as single cell RNA sequencing and imaging clarifying cell identity and cell-to-cell communication in situ, will further dissect the pathophysiology of AITL. Such innovative research will eventually provide new treatment modalities, with which we can control this intractable disease better.

## Supplementary information

Supplemental Table 1
